# Bayes factor functions for reporting outcomes of hypothesis tests

**DOI:** 10.1073/pnas.2217331120

**Published:** 2023-02-13

**Authors:** Valen E. Johnson, Sandipan Pramanik, Rachael Shudde

**Affiliations:** ^a^Department of Statistics, Texas A&M University, College Station, TX 77843-3143

**Keywords:** Bayes factors, meta-analysis, *P*-value, replication study, significance threshold

## Abstract

Bayes factors represent a useful alternative to *P*-values for reporting outcomes of hypothesis tests by providing direct measures of the relative support that data provide to competing hypotheses. Unfortunately, the competing hypotheses have to be specified, and the calculation of Bayes factors in high-dimensional settings can be difficult. To address these problems, we define Bayes factor functions (BFFs) directly from common test statistics. BFFs depend on a single noncentrality parameter that can be expressed as a function of standardized effects, and plots of BFFs versus effect size provide informative summaries of hypothesis tests that can be easily aggregated across studies. Such summaries eliminate the need for arbitrary *P*-value thresholds to define “statistical significance.” Because BFFs are defined using nonlocal alternative prior densities, they provide more rapid accumulation of evidence in favor of true null hypotheses without sacrificing efficiency in supporting true alternative hypotheses. BFFs can be expressed in closed form and can be computed easily from *z*, *t*, *χ*^2^, and *F* statistics.

## Significance

Bayes factors represent an informative alternative to *P*-values for reporting outcomes of hypothesis tests. They provide direct measures of the relative support that data provide to competing hypotheses and are able to quantify support for true null hypotheses. However, their use has been limited by several factors, including the requirement to specify alternative hypotheses and difficulties encountered in their calculation. Bayes factor functions (BFFs) overcome these difficulties by defining Bayes factors from classical test statistics and using standardized effect sizes to define alternative hypotheses. BFFs provide clear summaries of the outcome from a single experiment, eliminate arbitrary significance thresholds, and are ideal for combining evidence from replicated studies.

Two approaches are commonly used to summarize evidence from statistical hypothesis tests: *P*-values and Bayes factors. *P*-values are more frequently reported. As noted in the American Statistical Association Statement on Statistical Significance and *P*-Values, the significance of many published scientific findings is based on *P*-values, even though this index “is commonly misused and misinterpreted. This has led to some scientific journals discouraging the use of *P*-values, and some scientists and statisticians recommending their abandonment ... Informally, a *P*-value is the probability under a specified statistical model that a statistical summary of the data would be equal to or more extreme than its observed value” (1). *P*-values do not provide a direct measure of support for either the null or alternative hypotheses, and their use in defining arbitrary thresholds for defining statistical significance has long been a subject of intense debate; see, for example, refs. 2–8. Interpreting evidence provided by *P*-values across replicated studies is also challenging.

Bayes factors represent the ratio of the marginal probability assigned to data by competing hypotheses and, when combined with prior odds assigned between hypotheses, yield an estimate of the posterior odds that each hypothesis is true. That is,

[1] $$\begin{array}{c} \;\text{posterior odds}=\;\text{Bayes factor}\times \;\text{prior odds}, \end{array}$$

or, more precisely,

[2] $$\begin{array}{c} \frac{P(H_{1}|x)}{P(H_{0}|x)}=\frac{m_{1}\left(x\right)}{m_{0}\left(x\right)}\times \frac{P(H_{1})}{P(H_{0})}. \end{array}$$

Here, $P(H_{i}|x)$ denotes the posterior probability of hypothesis *H*_*i*_ given data x; *P*(*H*_*i*_) denotes the prior probability assigned to *H*_*i*_, and $m_{i}\left(x\right)$ denotes the marginal probability (or probability density function) assigned to the data under hypothesis *H*_*i*_, for *i* = 0 (null) or *i* = 1 (alternative).

The marginal density of the data under the alternative hypothesis is given by

[3] $$\begin{array}{c} m_{1}\left(x\right)=\int_{\Theta }f\left(x|\theta \right)\pi_{1}\left(\theta \right)d\;\theta , \end{array}$$

where f(x|θ) denotes the sampling density of the data given an unknown parameter *θ*. In null hypothesis significance tests (NHSTs), the marginal density of the data under the null hypothesis, $m_{0}\left(x\right)$, is simply the sampling density of the data assumed under the null hypothesis. That is, if *H*_0_ : *θ* = *θ*_0_, then $m_{0}\left(x\right)=f\left(x|\theta_{0}\right)$. The function *π*_1_(*θ*) represents the prior density for the parameter of interest *θ* under the alternative hypothesis, i.e., the alternative prior density.

The specification of *π*_1_(*θ*) is problematic. As a consequence, numerous Bayes factors based on “default” alternative prior densities have been proposed. Among others, these include (9–16). Nonetheless, the value of a Bayes factor depends on the alternative prior density used in its definition, and it is generally difficult to justify or interpret any single default choice. In addition, the numerical calculation of Bayes factors can be difficult, often requiring specialized software, and each of these problems is exacerbated in high-dimensional settings, e.g., ref. 17.

We propose several modifications to the existing Bayes factor methodology designed to enhance the report of scientific findings. First, we define Bayes factors directly from standard *z*, *t*, *χ*^2^, and *F* test statistics (18). Under the null hypotheses, the distribution of these test statistics is known. Under alternative hypotheses, the asymptotic distributions of these test statistics depend only on scalar-valued noncentrality parameters. Thus, the specification of the prior density that defines the alternative hypothesis is simplified, and no prior densities need to be specified under the null hypothesis.

Second, for a given test statistic, we calculate a range of Bayes factors by varying the prior densities imposed on the noncentrality parameter used to define the alternative hypothesis. The families of prior densities used to define Bayes factors are indexed by standardized effect size. Bayes factor functions (BFFs) are defined as the mapping of standardized effects to Bayes factors (or, more formally, the mapping of prior densities centered on standardized effect sizes to Bayes factors). BFFs thus make the connection between Bayes factors and prior assumptions more transparent by allowing Bayes factors to be interpreted in the context of the prior densities used in their definition. Because BFFs provide Bayes factors as a function of standardized effect size, they also facilitate the integration of evidence across multiple studies of the same phenomenon.

Third, the prior densities we propose for noncentrality parameters are special cases of nonlocal alternative prior (NAP) densities. These densities are identically zero when the noncentrality parameter is zero. This property makes it possible to more quickly accumulate evidence in favor of both true null and true alternative hypotheses (19–21); this particular feature of NAP densities is discussed in detail in ref. 22.

Finally, we provide closed-form expressions for Bayes factors and BFFs. These expressions eliminate computational difficulties sometimes encountered when calculating other Bayes factors.

## 1. Mathematical Framework

We write *a*|**b** ∼ *D*(**b**) to indicate that a random variable *a* has distribution *D* that depends on a parameter vector **b**. *N*(*a*, *b*) denotes the normal distribution with mean *a* and variance *b*; *T*(*ν*, *λ*) denotes a *t* distribution with *ν* degrees-of-freedom and noncentrality parameter *λ*; *F*(*k*, *m*, *λ*) denotes an *F* distribution on *k*, *m* degrees-of-freedom and noncentrality parameter *λ*; *G*(*α*, *λ*) denotes a gamma random variable with shape parameter *α* and rate parameter *λ*; *H*(*ν*, *λ*) denotes a *χ*^2^ distribution on *ν* degrees of freedom and noncentrality parameter *λ*; and *J*(*μ*_0_, *τ*^2^) denotes a normal moment distribution with mean *μ*_0_ and rate parameter *τ*^2^ (19). The density of a *J*(*μ*_0_, *τ*^2^) random variable can be written as

[4] $$\begin{array}{c} j\left(x|\mu_{0},\tau^{2}\right)=\frac{{\left(x-\mu_{0}\right)}^{2}}{\sqrt{2\pi }\tau^{3}}\exp\left(-\frac{{\left(x-\mu_{0}\right)}^{2}}{2\tau^{2}}\right). \end{array}$$

The modes of this distribution occur at $x=\mu_{0}\pm \sqrt{2}\tau$.

We use lowercase letters to denote corresponding densities; e.g., a gamma density evaluated at *x* is written *g*(*x*|*α*, *λ*).

With this notation, theorems describing Bayes factors based on *z*, *t*, *F*, and *χ*^2^ statistics are provided below. Each Bayes factor depends on a hyperparameter *τ*^2^ and is denoted by *BF*_10_(*x*|*τ*^2^). Procedures for setting *τ*^2^ as a function of standardized effect size are described in *Bayes Factors as Functions of Standardized Effect Size*. Proofs of the theorems appear in (*SI Appendix*).

Theorem 1. ***z test.** Assume that the distributions of a random variable z under the null and alternative hypotheses are described by*

[5] $$H_{0}:z∼N\left(0,1\right),$$

[6] $$H_{1}:z|\lambda ∼N\left(\lambda ,1\right),\;\lambda |\tau^{2}∼J\left(0,\tau^{2}\right).$$

*Then, the Bayes factor in favor of the alternative hypothesis is*

[7] $$\begin{array}{cc} BF_{10}\left(z|\tau^{2}\right)= & \;{\left(\tau^{2}+1\right)}^{-3/2}\left(1+\frac{\tau^{2}z^{2}}{\tau^{2}+1}\right)\\ & \times \exp\left[\frac{\tau^{2}z^{2}}{2(\tau^{2}+1)}\right]. \end{array}$$

Theorem 2. ***t test.** Assume that the distributions of a random variable t under the null and alternative hypotheses are described by*

[8] $$H_{0}:t∼T\left(\nu ,0\right),$$

[9] $$H_{1}:t|\lambda ∼T\left(\nu ,\lambda \right),\;\lambda |\tau^{2}∼J\left(0,\tau^{2}\right).$$

*Then, the Bayes factor in favor of the alternative hypothesis is*

[10] $$\begin{array}{c} BF_{10}\left(t|\tau^{2}\right)={\left(\tau^{2}+1\right)}^{-3/2}{\left(\frac{r}{s}\right)}^{\frac{\nu +1}{2}}\left(1+\frac{qt^{2}}{s}\right), \end{array}$$

*where*

$$\begin{array}{c} r=1+\frac{t^{2}}{\nu },\;s=1+\frac{t^{2}}{\nu (1+\tau^{2})},\;\mathrm{and}\;q=\frac{\tau^{2}\left(\nu +1\right)}{\nu (1+\tau^{2})}. \end{array}$$

Theorem 3. **$\chi^{2}$ *test.*** *Assume that the distributions of a random variable *h* under the null and alternative hypotheses are described by*

[11] $$H_{0}:h∼H\left(k,0\right),$$

[12] $$H_{1}:h|\lambda ∼H\left(k,\lambda \right),\;\lambda |\tau^{2}∼G\left(\frac{k}{2}+1,\frac{1}{2\tau^{2}}\right).$$

*Then, the Bayes factor in favor of the alternative hypothesis is*

[13] $$\begin{array}{cc} BF_{10}\left(h|\tau^{2}\right)= & \;{\left(\tau^{2}+1\right)}^{-k/2-1}\left(1+\frac{\tau^{2}}{k(\tau^{2}+1)}h\right)\\ & \times \exp\left(\frac{\tau^{2}h}{2(\tau^{2}+1)}\right). \end{array}$$

For *k* = 1 and *z*^2^ = *h*, the Bayes factor in Eq. **13** has the same value as the Bayes factor specified in Eq. **7**. The choice of the shape parameter as *k*/2 + 1 for the gamma density (a scaled *χ*_*k* + 2_^2^ random variable) in Eq. **12** was based on the fact that *χ*_*ν*_^2^ distributions are not 0 at the origin for integer degrees of freedom *ν* < 3. Thus, they are not NAP densities for *ν* < 3 and typically cannot provide strong evidence in favor of true null hypotheses without large sample sizes (19).

Theorem 4. ***F test.** Assume that the distributions of a random variable *f* under the null and alternative hypotheses are described by*

[14] $$H_{0}:f∼F\left(k,m,0\right),$$

[15] $$H_{1}:f|\lambda ∼F\left(k,m,\lambda \right),\;\lambda |\tau^{2}∼G\left(\frac{k}{2}+1,\frac{1}{2\tau^{2}}\right).$$

*Then, the Bayes factor in favor of the alternative hypothesis is*

[16] $$\begin{array}{cc} BF_{10}\left(f|\tau^{2}\right)= & \;{\left(\tau^{2}+1\right)}^{-\frac{k}{2}-1}{\left[\frac{\left(1+\frac{\mathrm{kf}}{m}\right)}{\left(1+\frac{\mathrm{kf}}{v}\right)}\right]}^{\frac{k+m}{2}}\\ & \times \left[1+\frac{\;\left(k+m\right)\tau^{2}\;f}{v\;\;\left(1+\frac{\mathrm{kf}}{v}\right)}\right], \end{array}$$

*where v* = *m*(*τ*^2^ + 1).

For *k* = 1 and *t*^2^ = *f*, the Bayes factors in Eq. **16** has the same value as the Bayes factor specified in Eq. **10**.

### A. Bayes Factors as Functions of Standardized Effect Size.

Theorems 1–4 describe Bayes factors based on classical test statistics. Like other Bayes factors, these Bayes factors depend on a hyperparameter *τ*^2^. Rather than ignoring this dependence and simply reporting a single Bayes factor, we construct BFFs that vary with *τ*^2^. Unfortunately, *τ*^2^ is difficult to interpret scientifically, and, as we show below, its interpretation changes from one test to another. For this reason, we report BFFs as functions of standardized effect sizes.

To illustrate this procedure, consider a *z* test of a null hypothesis *H*_0_ : *μ* = 0 based on a random sample *x*_1_, …, *x*_*n*_, where *x*_*i*_ ∼ *N*(*μ*, *σ*) and *σ*^2^ is known. For this test, $z=\sqrt{n}\overset{¯}{x}/\sigma$, and the distribution of *z* is

[17] $$\begin{array}{c} z|\mu ,\sigma ∼N\left(\frac{\sqrt{n}\mu }{\sigma },1\right). \end{array}$$

Under the null hypothesis, *z* ∼ *N*(0, 1). Under the alternative hypothesis, *μ* defines the deviation from the null value 0 and is called the effect size. The noncentrality parameter is $\lambda \;=\;\sqrt{n}\mu /\sigma$.

Effect sizes are often standardized. Standardization can serve two purposes. First, it makes the effect size invariant to units of measurement—for example, whether weights are measured in ounces or grams. Second, standardization scales the effect size according to the random variation between observational units. For the *z* test, the effect size *μ* can be standardized by dividing it by the SD of the observations, leading to a standardized effect *ω* = *μ*/*σ*. The noncentrality parameter for the test can then be expressed as $\lambda =\sqrt{n}\omega$. Cohen categorizes standardized effect sizes as small (0.2), medium (0.5), and large (0.8), and effect sizes larger 1.0 are not common in the social and behavioral sciences (23, 24).

Given a relationship between a standardized effect size *ω* and a noncentrality parameter *λ*, we compute the BFF by setting *τ*^2^ so that the modes of the prior density on *λ* occur at values defined by standardized effect sizes *ω*.

More explicitly, suppose that the noncentrality parameter *λ* can be written as a function of the standardized effect size *ω* as *λ* = *r*(*ω*), and let *π*(*λ*|*τ*^2^) denote a generic prior density on *λ* given *τ*^2^. For given *ω*, *τ*_*ω*_^2^ is implicitly defined such that,

[18] $$\begin{array}{c} r\left(\omega \right)=\underset{\lambda }{\arg\max}\;\pi \left(\lambda |\tau_{\omega }^{2}\right), \end{array}$$

the value of *τ*^2^ that makes the prior modes equal to *r*(*ω*). Given *τ*_*ω*_^2^ for a range of *ω* values, the BFF based on *x* consists of ordered pairs (*BF*(*x*|*τ*_*ω*_^2^),*ω*).

To illustrate this procedure, consider again the test of a normal mean. The noncentrality parameter for this test is $\lambda =\sqrt{n}\omega$. The default prior on the noncentrality parameter *λ* is a *J*(0, *τ*^2^) distribution, which has maxima at $\lambda^{*}=\pm \sqrt{2}\tau$ (that is, $\pm \sqrt{2}\tau =\underset{\lambda }{\arg\max}\;j\left(\lambda |0,\tau^{2}\right)$). Equating the noncentrality parameter to the modes of the prior density (i.e., $\sqrt{n}\omega =\lambda^{*}=\pm \sqrt{2}\tau$) implies that *τ*_*ω*_^2^ = *nω*^2^/2.

**Fig. 1.**
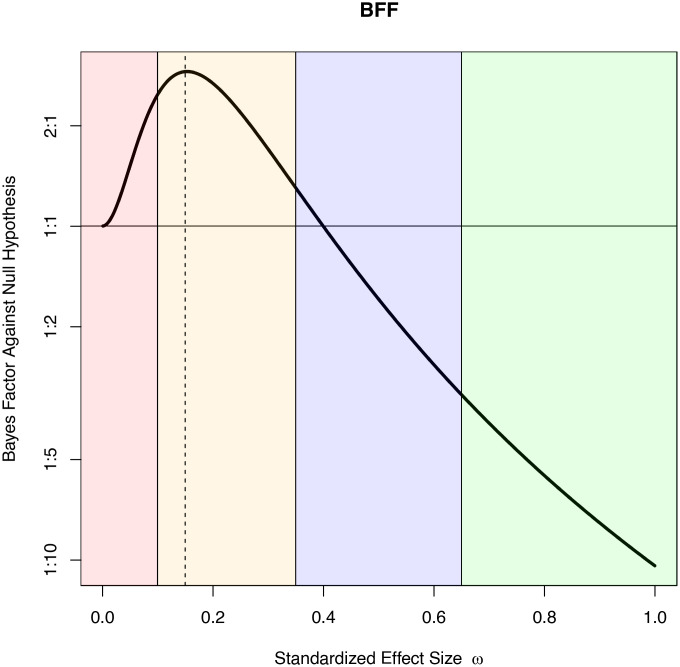
Plot of the BFF, *BF*_10_(2|100*ω*^2^/2), against *ω* for a *z* test with *z* = 2 and *n* = 100. Bayes factors are displayed as odds in favor of the alternative hypothesis. The vertical axis is displayed on the logarithmic scale. The vertical dotted line indicates that the maximum Bayes factor of 2.90 corresponds to a standardized effect size of 0.15. The horizontal line at 1 : 1 denotes a Bayes factor of 1.0.

Fig. 1 displays the BFF in favor of the alternative hypothesis using the mapping *τ*_*ω*_^2^ = *nω*^2^/2 when *z* = 2.0 and *n* = 100. From the BFF, we can conclude that the maximum Bayes factor in favor of the alternative hypothesis is 2.90 and that this Bayes factor is achieved when the prior modes on the standardized effect size are ±0.15. That is, the value of 2.9 is obtained conditionally on the (data-dependent) selection of *τ*_*ω*_^2^ = 1.125 and a *J*(0, 1.125) alternative prior on the noncentrality parameter *λ*. More generally, the selection of the maximum Bayes factor from the BFF provides the strongest information in favor of the alternative hypothesis that can be obtained from within the specified family of prior densities on the noncentrality parameter.

The odds in favor of alternative hypotheses fall below 1:1 when the alternative hypotheses are centered on standardized effect sizes greater than 0.4; they fall below 1:5 when the prior modes of the alternative prior density on standardized effect size are greater than 0.8. Other examples of BFFs for *z* statistics are provided in *SI Appendix*.

As the standardized effect size approaches 0, the alternative hypothesis becomes indistinguishable from the null hypothesis, driving the Bayes factor to 1. The red, orange, blue, and green zones in this figure are arbitrarily colored and correspond to very small ((0, 0.1)), small ((0.1, 0.35)), medium ((0.35, 0.65)), and large (> 0.65) standardized effect sizes, respectively.

Table 1 provides a mapping between standardized effect sizes *ω* and *τ*_*ω*_^2^ for several common statistical tests. Special cases of tests in the “Multinomial/Counts” row include Pearson’s *χ*^2^ test for goodness of fit (*s* = 0 and f(θ) known) and tests for independence in contingency tables (*K* − *s* − 1 = (# rows  −  1)(#columns − 1)). Recall that a test for the value of a binomial proportion can be framed as Pearson’s *χ*^2^ test. In contrast, a test for a difference in proportions can be specified as a test for independence in contingency tables.

**Table 1.** Default choices for *τ*_*ω*_^2^

Test	Statistic	Standardized effect (*ω*)	*τ*_*ω*_^2^
1-sample z	$\frac{\sqrt{n}\overset{¯}{x}}{\sigma }$	$\frac{\mu }{\sigma }$	$\frac{n\omega^{2}}{2}$
1-sample t	$\frac{\sqrt{n}\overset{¯}{x}}{s}$	$\frac{\mu }{\sigma }$	$\frac{n\omega^{2}}{2}$
2-sample z	$\frac{\sqrt{n_{1}n_{2}}\left({\overset{¯}{x}}_{1}-{\overset{¯}{x}}_{2}\right)}{\sigma \sqrt{n_{1}+n_{2}}}$	$\frac{\mu_{1}-\mu_{2}}{\sigma }$	$\frac{n_{1}n_{2}\omega^{2}}{2(n_{1}+n_{2})}$
2-sample t	$\frac{\sqrt{n_{1}n_{2}}\left({\overset{¯}{x}}_{1}-{\overset{¯}{x}}_{2}\right)}{s\sqrt{n_{1}+n_{2}}}$	$\frac{\mu_{1}-\mu_{2}}{\sigma }$	$\frac{n_{1}n_{2}\omega^{2}}{2(n_{1}+n_{2})}$
Multinomial/Poisson	$\chi_{\nu }^{2}=\sum_{i=1}^{k}\frac{{\left(n_{i}-nf_{i}\left(\hat{\theta }\right)\right)}^{2}}{nf_{i}\left(\hat{\theta }\right)}$	${\left(\frac{p_{i}-f_{i}\left(\theta \right)}{\sqrt{f_{i}\left(\theta \right)}}\right)}_{k\times 1}$	$\frac{n\omega \prime \omega }{k}=n{\tilde{\omega }}^{2}$
Linear model	$F_{k,n-p}=\frac{(RSS_{0}-RSS_{1})/k}{\left[\left(RSS_{1}\right)/\left(n-p\right)\right]}$	$\frac{L^{-1}\left(A\beta -a\right)}{\sigma }$	$\frac{n\omega \prime \omega }{2k}=\frac{n{\tilde{\omega }}^{2}}{2}$
Likelihood ratio	$\chi_{k}^{2}=-2\log\left[\frac{l(\theta_{r0},\hat{\theta_{s}})}{l(\hat{\theta })}\right]$	$L^{-1}\left(\theta_{r}-\theta_{r0}\right)$	$\frac{n\omega \prime \omega }{k}=n{\tilde{\omega }}^{2}$

For one-sample tests, *x*_1_, …, *x*_*n*_ are assumed to be iid *N*(*μ*, *σ*^2^), where *n* refers to sample size. In two-sample tests, *x*_*j*, 1_, …, *x*_*j*, *n*_*j*__, *j* = 1, 2, are assumed to be iid *N*(*μ*_*j*_, *σ*^2^). Integers *n*_1_ and *n*_2_ refer to sample sizes in each group. A bar over a variable denotes the sample mean. The variance of normal observations is denoted by *σ*^2^ and is assumed to be equal in both groups in two-sample tests. Standard deviations are denoted by *s* and are the pooled estimate in the two-sample *t* test. In multinomial/Poisson tests, f(θ) maps an *s* × 1 vector θ into a *k* × 1 probability vector, where *k* denotes the number of cells. The degrees of freedom *ν* equals *k* − *s* − 1. The quantities *p*_*i*_ and *n*_*i*_ represent cell probabilities and counts, respectively, and *n* is the sum of all cell counts. In the linear model, the alternative hypothesis is Aβ=a, where **A** is a *k* × *p* matrix of rank *k*, β is a *p* × 1 vector of regression coefficients, and **a** is a *k* × 1 vector. The quantities *RSS*_0_ and *RSS*_1_ denote the residual sum of squares under the null and alternative hypotheses, respectively. The quantity *n* is the number of observations, and *σ*^2^ is the observational variance. In the likelihood ratio test, *l*(⋅) denotes the likelihood function for a parameter vector $\theta =(\theta_{r},\theta_{s})$. The *k* × 1 subvector $\theta_{r}$ equals $\theta_{r0}$ under the null hypothesis. The maximum likelihood estimate of θ under the alternative hypothesis is $\hat{\theta }$, and the maximum likelihood of $\theta_{s}$ under the null hypothesis is ${\hat{\theta }}_{s}$. In the linear model and likelihood ratio tests, the matrix **L**^−1^ represents the Cholesky decomposition of the covariance matrix for the tested parameters, scaled to a single observation. Further explanation of *τ*_*ω*_^2^ values appear in *SI Appendix*.

The last three rows in Table 1 contain vectors of standardized effects ω. Because the recommended value of *τ*_*ω*_^2^ depends on ω only through the inner product ω′ω, in many applications, it is easier to study the BFF as a function of the root mean square effect size (RMSES), $\tilde{\omega }$, defined as

[19] $$\begin{array}{c} \tilde{\omega }=\sqrt{\frac{1}{k}\sum_{i=1}^{k}\omega_{i}^{2}}. \end{array}$$

The last entry in Table 1 provides default choices for *τ*_*ω*_^2^ based on the asymptotic distribution of the likelihood ratio statistic. This choice is based on classical results summarized in, for example, ref. 25. This entry is of particular interest due to the widespread application of the likelihood ratio test statistic in nonlinear models.

Justification for the values of *τ*_*ω*_^2^ in Table 1 appears in *SI Appendix*.

## 2. Applications

The following examples show how BFFs can be used to summarize outcomes of hypothesis tests based on *χ*^2^ and *F* test statistics.

### A. Cancer Sites and Blood Type Association.

White and Eisenberg (26) collected data from 707 patients with stomach cancer and investigated the association between cancer site and blood type. Data from their study are summarized in Table 2. The *χ*^2^ test for independence for these data is 12.65 on 6 degrees of freedom (*P* = 0.049).

**Table 2.** White and Eisenberg’s classification of cancer patients

	Results for the following blood groups		
Site	O	A	B or AB
Pylorus and antrum	104	140	52
Body and fundus	116	117	52
Cardia	28	39	11
Extensive	28	12	8

Bayes factors to test the independence of cancer site and blood type were previously calculated by refs. 27, 28, and 18. The Bayes factors reported in refs. 27 and 28 require the specification of prior distributions on the marginal probabilities of blood type and cancer site under the null hypothesis and the specification of a Dirichlet distribution on all combinations of blood type × cancer site probabilities under an alternative model. Johnson (18) maximized a Bayes factor based on the chi-squared statistic similar to that proposed in Theorem 3, except that the prior on the noncentrality parameter was a scaled chi-squared distribution on 6 degrees of freedom (rather than 8). The scale of the chi-squared prior was chosen to maximize the Bayes factor against the null hypothesis of independence. The Bayes factors reported in refs. 18, 27, and 28 were 2.97, 3.02, and 3.06, respectively.

**Fig. 2.**
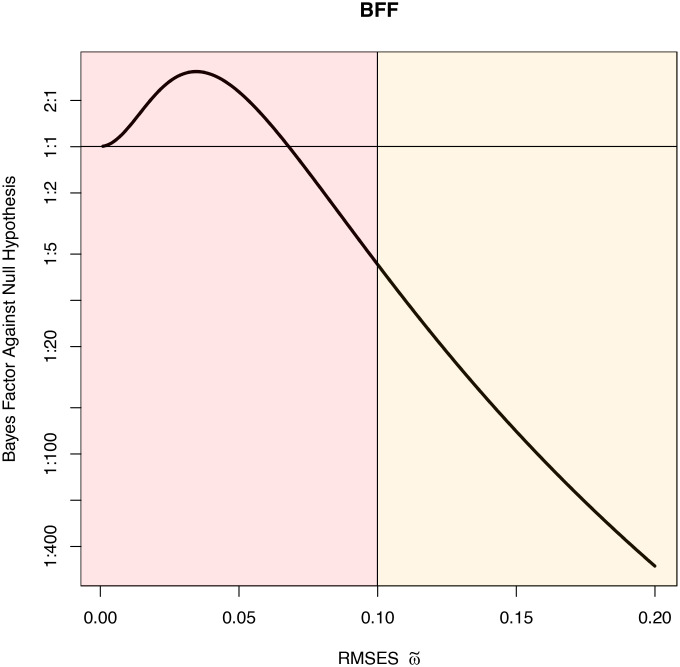
Plot of the BFF, $BF_{10}\left(12.65|707{\tilde{\omega }}^{2}\right)$, against $\tilde{\omega }$ for a *χ*_6_^2^ test with *h* = 12.65. Bayes factors are displayed as odds in favor of dependence between patient blood type and cancer site. The vertical axis is displayed on the logarithmic scale. The color coding is consistent with Fig. 1. The horizontal line in the plot corresponds to a Bayes factor of 1.0 (odds of 1:1).

Fig. 2 displays the BFF as a function of the RMSES using results from Theorem 3 and the *τ*_*ω*_^2^ values provided in Table 1. The maximum Bayes factor in favor of dependence occurs for $\tilde{\omega }=0.035$, where it equals 3.07. The Bayes factor favors the independence model against alternatives with $\tilde{\omega }>0.068$. A standardized effect size of 0.2 represents what is often considered a small standardized effect in the social science and medical literature (23), and the Bayes factor against such an effect size for these data is greater than 400:1. The Bayes factors reported in refs. 18, 27, and 28 and the maximum value of 3.07 reported here are, for many practical purposes, similar in their scientific interpretation. They all suggest that the data support an alternative hypothesis of nonindependence three times more than the null hypothesis of independence. However, the previous methods do not emphasize that evidence against the null hypothesis is garnered only for alternative hypotheses representing very small effect sizes ($\tilde{\omega }<0.068$), and even for such small effect sizes, the evidence is weak.

### B. Biases Associated with Confirmatory Information Processing.

To illustrate BFFs based on *F* statistics in replicated studies, we turn to a study reported in ref. 29 that was replicated in ref. 30. Both studies sought to determine whether states of self-regulation depletion or ego threat caused participants to exhibit more bias in confirmatory information processing. The studies compared preferences for decision-consistent and decision-inconsistent information processing between three groups: high depletion of self-regulation, low depletion of self-regulation, and ego-threatened subjects. The dependent variable consisted of a normalized score for participants’ selection of decision-consistent and decision-inconsistent reports regarding a hiring decision upon which they had made a preliminary decision. The original study’s authors recruited 85 undergraduate students as subjects, while 140 subjects participated in the replicated study. Differences between the outcomes in the three groups were assessed using one-way ANOVA. The *F* statistics reported in the original and replicated studies were *F*_2, 82_ = 4.05 (*P* = 0.021) and *F*_2, 137_ = 1.99 (*P* = .141), respectively.

To construct a Bayes factor from these studies, it is necessary to define the hypotheses being tested. To make the discussion more general, we assume a total of *S* studies; *S* = 2 in this example.

Let *x*_1_, …, *x*_*S*_ denote *S* independent *F* statistics with numerator degrees of freedom *k*_1_, …, *k*_*S*_ and denominator degrees of freedom *m*_1_, …, *m*_*S*_, respectively. In the present case, *k*_1_ = *k*_2_ = 2, and *m*_1_ = *n*_1_ − 3 and *m*_2_ = *n*_2_ − 3, where *n*_1_ = 85 and *n*_2_ = 140 are the sample sizes in the two studies.

Under the null hypothesis, we assume

[20] $$\begin{array}{c} H_{0}:x_{s}∼F\left(k_{s},m_{s},0\right),\;\;\text{for}\;s=1,\cdots ,S. \end{array}$$

Given the independence of the {*x*_*s*_}, the marginal density of the data under the null hypothesis is

[21] $$\begin{array}{c} m_{0}\left(x_{1},\cdots ,x_{S}\right)=\prod_{s=1}^{S}m_{0}\left(x_{s}\right)=\prod_{s=1}^{S}f\left(x_{s}|k_{s},m_{s},0\right). \end{array}$$

Under the alternative hypothesis, we assume

[22] $$\begin{array}{cc} H_{1}:x_{s}|\lambda ∼F\left(k_{s},m_{s},\lambda_{s}\right), & \;\lambda_{s}|\tau_{s}^{2}∼G\left(\frac{k_{s}}{2}+1,\frac{1}{2\tau_{s}^{2}}\right),\\ & \tau_{s}^{2}=\frac{n_{s}{\tilde{\omega }}^{2}}{2}. \end{array}$$

Different prior distributions are specified for the noncentrality parameters {*λ*_*s*_} to account for the dependence of these parameters on each study’s sample size. However, the rate parameters *τ*_*s*_^2^ that define these prior densities were determined from a common RMSES, $\tilde{\omega }$. This stipulation models the belief that the interventions have similar effects across studies.

Assuming that the noncentrality parameters are conditionally independent across studies, it follows that the marginal density of the data under the alternative hypothesis is

[23] $$\begin{array}{c} m_{1}\left(x_{1},\cdots ,x_{S}|\tilde{\omega }\right)=\prod_{s=1}^{S}m_{1}\left(x_{s}|\tau_{\tilde{\omega }}^{2}\right). \end{array}$$

Here, the dependence of the marginal densities on the assumed value of $\tilde{\omega }$ and $\tau_{\tilde{\omega }}^{2}$ has been indicated. Dividing equation (**23**) by (**21**) leads to

[24] $$\begin{array}{cc} BF_{10}\left(x_{1},\cdots ,x_{S}|\tilde{\omega }\right) & =\frac{\prod_{s=1}^{S}m_{1}\left(x_{s}|\tau_{\tilde{\omega }}^{2}\right)}{\prod_{s=1}^{S}m_{0}\left(x_{s}\right)}\\ & =\prod_{s=1}^{S}BF_{10}\left(x_{s}|\tau_{\tilde{\omega }}^{2}\right). \end{array}$$

Thus, the Bayes factor for the combined study can be obtained by multiplying the Bayes factors from the individual studies.

Eq. **24** can be applied generally to obtain Bayes factors based on independent *z*, *t*, *χ*^2^, and *F* statistics using Theorems 1–4 and Table 1, under the assumption that noncentrality parameters are drawn independently from their prior distributions.

Returning to our example, the Bayes factors for the two studies can be combined according to Eq. **24** and Theorem 4 as follows:

[25] $$\begin{array}{cc} BF_{10}[f_{2,82} & =4.05,f_{2,137}=1.99|\tilde{\omega }]\\ & =BF_{10}\left[f_{2,82}=4.05|\tau_{\tilde{\omega }}^{2}=\frac{85{\tilde{\omega }}^{2}}{2}\right]\\ & \;\times BF_{10}\left[f_{2,137}=1.99|\tau_{\tilde{\omega }}^{2}=\frac{140{\tilde{\omega }}^{2}}{2}\right]. \end{array}$$

**Fig. 3.**
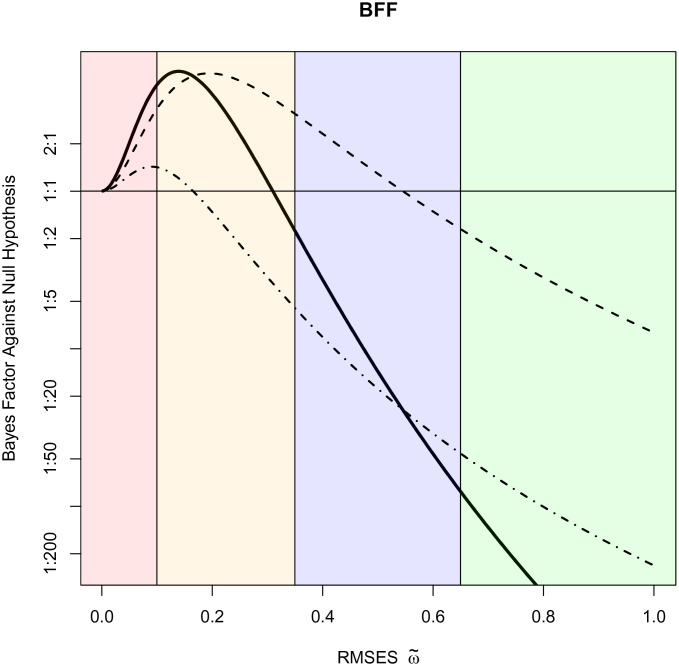
BFFs for confirmatory information processing studies. The upper dotted line depicts the BFF from the original study; the lower dotted line is for the replicated study. The solid line represents the BFF obtained by multiplying the Bayes factors from the two studies.

Fig. 3 depicts the BFF versus RMSES $\tilde{\omega }$ based on both experiments alongside the original and replication studies. By combining information from the two studies, we see that there is support for very small or small standardized effect sizes, with greater than 2:1 support for RMSES greater than 0.05 and less than 0.26. There is no support for standardized effect sizes greater than 0.31. The maximum Bayes factor against the null hypothesis of no effect was obtained for an RMSES of 0.14, where it was 5.75:1 against the null hypothesis of no group effect.

## 3. Discussion

The Bayes factors and BFFs described above are based on the specification of normal-moment and gamma prior densities imposed on scalar noncentrality parameters. Other prior specifications on noncentrality parameters are, of course, possible. However, the proposed prior densities possess several attractive features. Among these, they represent NAP densities, making it possible to accumulate evidence more rapidly in favor of true null hypotheses. They also yield closed-form expressions for Bayes factors, which facilitates BFF calculation. The coefficients of variation of the gamma priors in Theorems 3 and 4 are equal to $\sqrt{2/(k+2)}$ and so depend only on the (numerator) degrees of freedom of the test statistics. Under the proposed framework, the standard deviations of prior distributions on noncentrality parameters are thus scaled according to sample size. When used in conjunction with the normal moment priors specified in Theorems 1 and 2, these choices also yield Bayes factors that are invariant to the choice of the test statistic in the sense that *z* and *z*^2^ = *χ*_1_^2^ tests and *t*_*ν*_ and *t*_*ν*_^2^ = *F*_1, *ν*_ tests produce the same Bayes factors when a common value of *τ*^2^ is selected.

For the test statistics considered above, it is possible to compute a “maximum BFF” as the ratio of that test statistic’s noncentral alternative density to its central density under the null hypothesis (without averaging over a prior density). This procedure essentially produces a plot of the likelihood ratio for each test statistic. Because the probability that the test statistic matches this maximum value is either zero (continuous data) or small (discrete data), the maximum Bayes factor reported from such a procedure overstates evidence in favor of the alternative hypothesis. This procedure also precludes the collection of evidence in favor of true null hypotheses and fails to model the variability of standardized effect sizes across studies.

To a lesser extent, similar concerns also affect the interpretation of the maximum of the BFF defined using Theorems 1–4. However, these Bayes factors are obtained by averaging over prior densities and should be interpreted from a conditional perspective. For example, under the specified model assumptions, an appropriate interpretation of the data collected to study confirmatory information processing biases is that the maximum Bayes factor against the null hypothesis is at most 5.75:1; this Bayes factor occurs for the prior density corresponding to a RMSES of 0.14; and the data do not support alternative hypotheses with priors centered on RMSES greater than 0.31. Bayes factors against the null hypothesis are greater than 2:1 for alternative hypotheses corresponding to RMSES between approximately 0.05 and 0.26. Along similar lines, in the study of associations between cancer sites and blood types, there is no support for alternative hypotheses centered on RMSES greater than 0.07, and there is greater than 400:1 support against alternatives representing even small standardized effect sizes ($\tilde{\omega }\geq 0.20$).

An advantage of the conditional approach inherent to BFFs is that they provide evidence supporting specific alternative hypotheses. This is important when subjective prior information regarding effect size magnitude is unavailable. By integrating the likelihood function with respect to parameter values most consistent with a given effect size, Bayes factors in favor of plausible alternative hypotheses are thus not adversely affected by default prior specifications that place significant mass on unrealistically large or small parameter values under the given hypotheses.

Many scientists now acknowledge the critical role that replication studies play in improving the reproducibility of scientific studies (31, 32). The final example demonstrates that BFFs provide a formal mechanism to combine information collected across replicated experiments using only reported test statistics. Their use thus provides a potential tool for enhancing the reproducibility of scientific research.

Finally, BFFs provide a viable alternative to the report of *P*-values. Using BFFs, researchers can quickly assess the level of support that data provide to alternative hypotheses centered on a range of standardized effect sizes as well as the scientific significance of those standardized effects.

An R package to calculate default BFFs for tests described in this article, “BFF,” is available for download at cran.r-project.org.

## Supplementary Material

Appendix 01 (PDF)

## Data Availability

All data described in the article are contained within the article. An R package to implement methods is available in CRAN at https://cran.r-project.org/web/packages/BFF/index.html.
