# Supplementary material for: Bayes factor functions for reporting outcomes of hypothesis tests
Source: Proc Natl Acad Sci U S A. 2023 Feb 13;120(8):e2217331120. doi: 10.1073/pnas.2217331120 (PMC9974512; doi:10.1073/pnas.2217331120)
Supplement: Supplementary file 1 — Appendix 01 (PDF) [file pnas.2217331120.sapp.pdf]

Supporting Information for

Bayes factor functions for reporting outcomes of hypothesis tests

Valen E. Johnson, Sandipan Pramanik and Rachael Shudde

Corresponding Author Valen E. Johnson.
E-mail: vejohnson@tamu.edu

This PDF file includes:

Fig. S1
SI References

Proofs of Theorems

Throughout, $m_0(\mathbf{x})$ and $m_1(\mathbf{x})$ denote marginal densities of data vectors $\mathbf{x}$ under the null and alternative hypotheses, respectively, $BF_{10}(\mathbf{x}) = m_1(\mathbf{x})/m_0(\mathbf{x})$, and τ^2 is treated as a constant. The proofs of the first two theorems are similar to the proofs provided in the supplementary material of (1).

Proof of Theorem 1. By assumption,

$$m_0(z) = c\exp\left(-z^2/2\right), \qquad \text{where} \qquad c = \frac{1}{\sqrt{2\pi}} \tag{1}$$

and

$$m_1(z\,|\,\tau^2) = \int_{-\infty}^{\infty} \frac{c}{\sqrt{2\pi\tau^3}}\lambda^2 \exp\left(-\frac{\lambda^2}{2\tau^2}\right)\exp\left(-\frac{(z-\lambda)^2}{2}\right)d\lambda \tag{2}$$

$$= \int_{-\infty}^{\infty} \frac{c}{\sqrt{2\pi\tau^3}}\lambda^2 \exp\left[-\frac{1}{2}\left(\frac{\lambda^2}{\tau^2}+(z-\lambda)^2\right)\right]d\lambda. \tag{3}$$

Letting $a = \tau^2/(1+\tau^2)$ and noting

$$\frac{\lambda^2}{\tau^2}+(z-\lambda)^2 = \frac{1}{a}(\lambda - az)^2 + \frac{az^2}{\tau^2}, \tag{4}$$

it follows that

$$m_1(z\,|\,\tau^2) = \int_{-\infty}^{\infty} \frac{c}{\sqrt{2\pi\tau^3}}\lambda^2 \exp\left\{-\frac{1}{2}\left[\frac{1}{a}(\lambda-az)^2+\frac{az^2}{\tau^2}\right]\right\}d\lambda \tag{5}$$

$$= \frac{\sqrt{a}c}{\tau^3}\exp\left(-\frac{az^2}{2\tau^2}\right)\int_{-\infty}^{\infty}\frac{1}{\sqrt{2\pi a}}\lambda^2\exp\left[-\frac{(\lambda-az)^2}{2a}\right]d\lambda. \tag{6}$$

The integral represents the second moment of a normal distribution with mean az and variance a. Thus

$$m_1(z\,|\,\tau^2) = \frac{\sqrt{a}c}{\tau^3}\left[a+(az)^2\right]\exp\left(-\frac{az^2}{2\tau^2}\right). \tag{7}$$

Substituting for a and dividing $m_1(z\,|\,\tau^2)$ by $m_0(z)$ produces the Bayes factor appearing in Theorem 1 of the article.

■

Proof of Theorem 2. Under H_0, the marginal density of the t statistic can be expressed

$$m_0(t) = \frac{\Gamma[(\nu+1)/2]}{\sqrt{\nu\pi}\Gamma(\nu/2)}\left(1+\frac{t^2}{\nu}\right)^{-(\nu+1)/2}. \tag{8}$$

Defining

$$c = \frac{\nu^{\nu/2}}{\sqrt{\pi}\Gamma(\nu/2)d^{\nu+1}2^{(\nu-1)/2}} \qquad \text{with} \qquad d = \sqrt{t^2+\nu}, \tag{9}$$

$m_1(t)$ can be expressed by integrating the integral form of the non-central t density from (2) with respect to a $J(0,\tau^2)$ prior density to obtain

$$m_1(t) = \int_{-\infty}^{\infty}\int_0^{\infty} c\exp\left(-\frac{\nu\lambda^2}{2d^2}\right)y^{\nu}\exp\left[-\frac{(y-\lambda t/d)^2}{2}\right]\frac{\lambda^2}{\sqrt{2\pi\tau^3}}\exp\left(-\frac{\lambda^2}{2\tau^2}\right)dy\,d\lambda. \tag{10}$$

Again letting $a = \tau^2/(\tau^2+1)$ and noting that

$$\frac{\nu\lambda^2}{d^2}+(y-\lambda t/d)^2+\frac{\lambda^2}{\tau^2} = \frac{1}{a}\left(\lambda - \frac{ayt}{d}\right)^2 + y^2\left(1-\frac{at^2}{d^2}\right), \tag{11}$$

application of Fubini's theorem implies that the integral with respect to λ is proportional to the second moment of a normal density with variance a and mean (ayt/d). Thus,

$$m_1(t) = \int_0^{\infty} cy^{\nu}\frac{1}{\tau^2\sqrt{\tau^2+1}}\left[a+\frac{(ayt)^2}{d^2}\right]\exp\left[-\frac{y^2}{2}\left(1-\frac{at^2}{d^2}\right)\right]dy. \tag{12}$$

Changing variables to $z = y^2$, it follows that equation (12) represents the weighted sum of two gamma functions. Define

$$b = 1 - \frac{at^2}{d^2}, \qquad \text{which implies} \qquad \frac{1}{b} = \frac{1 + \frac{t^2}{\nu}}{1 + \frac{t^2}{\nu(\tau^2+1)}}. \tag{13}$$

Integrating equation (12) leads to

$$m_1(t) = c\,\frac{1}{2(\tau^2+1)^{3/2}} \left[\frac{2^{(\nu+1)/2}\Gamma\left(\frac{\nu+1}{2}\right)}{b^{(\nu+1)/2}} + \frac{at^2 2^{(\nu+3)/2}\Gamma\left(\frac{\nu+3}{2}\right)}{d^2 b^{(\nu+3)/2}} \right] \tag{14}$$

$$= \frac{\nu^{\nu/2}}{\sqrt{\pi}\Gamma(\nu/2) d^{\nu+1}(\tau^2+1)^{3/2}} \left[\frac{\Gamma\left(\frac{\nu+1}{2}\right)}{b^{(\nu+1)/2}} + \frac{at^2(\nu+1)\Gamma\left(\frac{\nu+1}{2}\right)}{d^2 b^{(\nu+3)/2}} \right]. \tag{15}$$

Also,

$$\left(d\nu^{-1/2}\right)^{-\nu-1} = \left(1 + \frac{t^2}{\nu}\right)^{-(\nu+1)/2}. \tag{16}$$

Thus, dividing $m_1(t)$ in equation (15) by $m_0(t)$ in equation (8) leads to

$$BF_{10}(t) = (\tau^2+1)^{-3/2} b^{-(\nu+1)/2} \left[1 + \frac{a(\nu+1)t^2}{bd^2}\right]. \tag{17}$$

But $1/b = r/s$ and

$$\frac{a(\nu+1)t^2}{bd^2} = \frac{qt^2}{s}, \tag{18}$$

yielding the Bayes factor specified in the theorem.

■

Proof of Theorem 3. Under H_0, the density of h is

$$m_0(h) = \frac{1}{2^{k/2}\,\Gamma\left(\frac{k}{2}\right)} h^{\frac{k}{2}-1} \exp\left(-\frac{h}{2}\right). \tag{19}$$

Under H_1, the marginal density of h is obtained by integrating the non-central chi-squared density (2) with respect to the prior on the non-centrality parameter λ:

$$m_1(h) = \int_0^\infty \sum_{i=0}^{\infty} \frac{e^{-\lambda/2}\left(\frac{\lambda}{2}\right)^i}{i!\,2^{\frac{k+2i}{2}}\,\Gamma\left(\frac{k}{2}+i\right)} h^{\frac{k}{2}+i-1} \exp\left(-\frac{h}{2}\right) \tag{20}$$

$$\times \frac{\lambda^{\frac{k}{2}} \exp\left(-\frac{\lambda}{2\tau^2}\right)}{(2\tau^2)^{\frac{k}{2}+1}\Gamma\left(\frac{k}{2}+1\right)}\, d\lambda. \tag{21}$$

Noting that

$$\int_0^\infty \lambda^{\frac{k}{2}+i} \exp\left[-\lambda\left(\frac{1}{2} + \frac{1}{2\tau^2}\right)\right] d\lambda = \frac{\left(\frac{k}{2}+i\right)\Gamma\left(\frac{k}{2}+i\right)}{\left(\frac{\tau^2+1}{2\tau^2}\right)^{\frac{k}{2}+i+1}}, \tag{22}$$

application of Fubini's theorem leads to

$$m_1(h) = b(h) \sum_{i=0}^{\infty} \left(\frac{2\tau^2}{\tau^2+1}\right)^i \frac{\left(\frac{k}{2}+i\right)h^i}{2^{2i}\,i!}, \tag{23}$$

where

$$b(h) = \frac{1}{(\tau^2+1)^{\frac{k}{2}+1}\Gamma\left(\frac{k}{2}+1\right)2^{\frac{k}{2}}}\, h^{\frac{k}{2}-1} e^{-h/2}. \tag{24}$$

Because

$$\sum_{j=0}^{\infty} \frac{(c+j)a^j}{j!} = (c+a)e^a, \tag{25}$$

equation (23) can be rewritten as

$$m_1(h) = b(h)\left[\frac{k}{2} + \frac{\tau^2 h}{2(\tau^2+1)}\right] \exp\left(\frac{\tau^2 h}{2(\tau^2+1)}\right). \tag{26}$$

Noting that

$$\frac{b(h)}{m_0(h)} = (\tau^2+1)^{-(\frac{k}{2}+1)}\frac{2}{k} \qquad [27]$$

and dividing in equation (26) yields the Bayes factor stated in the theorem.

■

As an aside, the $m_1(h)$ can be expressed as

$$m_1(h) = \frac{1}{\tau^2+1} g\left(h\,;\frac{k}{2},\frac{1}{2(\tau^2+1)}\right) + \frac{\tau^2}{\tau^2+1} g\left(h\,;\frac{k}{2}+1,\frac{1}{2(\tau^2+1)}\right). \qquad [28]$$

Proof of Theorem 4. Under H_0, the marginal density of the f statistic is

$$m_0(f) = B\left(\frac{k}{2},\frac{m}{2}\right)^{-1}\left(\frac{k}{m}\right)^{k/2} f^{k/2-1}\left(1+\frac{k}{m}f\right)^{-(k+m)/2}, \qquad [29]$$

where $B(k/2, m/2) = \Gamma(k/2)\Gamma(m/2)/\Gamma[(k+m)/2]$ is the beta function. The marginal density of f under H_1 is obtained by integrating the non-central f density (2) with respect to the gamma prior on its non-centrality parameter λ:

$$m_1(f) \;=\; \int_0^\infty \left(\frac{k}{m}\right)^{k/2} e^{-\lambda/2}\sum_{r=0}^{\infty}\left(\frac{k\lambda}{2m}\right)^r \frac{1}{r!}B\left(\frac{k}{2}+r,\frac{m}{2}\right)^{-1} \qquad [30]$$

$$\times\; \frac{f^{r+k/2-1}}{(1+kf/m)^{r+(k+m)/2}}\,\frac{\lambda^{k/2}\exp(-\lambda/(2\tau^2))}{(2\tau^2)^{k/2+1}\Gamma(k/2)}\,d\lambda. \qquad [31]$$

Defining

$$a(f) = \frac{\left(\frac{k}{m}\right)^{k/2} f^{k/2-1}}{\Gamma(\frac{k}{2}+1)\Gamma(\frac{m}{2})(2\tau^2)^{1+k/2}\left(1+\frac{k}{m}f\right)^{-(k+m)/2}}, \qquad [32]$$

application of Fubini's theorem, recognizing

$$\Gamma\left(\frac{k}{2}+r\right)\left(\frac{1}{2}+\frac{1}{2\tau^2}\right)^{-r-k/2-1} = \int_0^\infty \lambda^{r+k/2}\exp\left[-\frac{\lambda}{2}\left(1+\frac{1}{\tau^2}\right)\right]d\lambda, \qquad [33]$$

and expanding $\Gamma(r+\frac{k}{2}+\frac{m}{2})$ as an integral allows the marginal density to be expressed as

$$m_1(f) \;=\; \int_0^\infty a(f)\,e^{-x}\,\frac{x^{k/2+m/2-1}}{\left(\frac{1}{2}+\frac{1}{2\tau^2}\right)} \qquad [34]$$

$$\times\; \sum_{r=0}^{\infty}\left(r+\frac{k}{2}\right)\left[\frac{kxf}{m\left(1+\frac{k}{m}f\right)\left(1+\frac{1}{\tau^2}\right)}\right]^r\frac{1}{r!}\,dx. \qquad [35]$$

The series in equation (35) can be summed and is equal to

$$\frac{k}{2}e^{b(x)} + b(x)e^{b(x)}, \qquad [36]$$

where $b(x)$ is the bracketed term raised to power r. Letting $c = b(x)/x$, the integration with respect to x represents the sum of two scaled gamma functions, leading to

$$m_1(f) = \frac{a(f)\,\Gamma\left(\frac{k+m}{2}\right)}{(1-c)^{\frac{k+m}{2}}}\left[\frac{2\tau^2}{\tau^2+1}\right]^{\frac{k}{2}}\left[\frac{k}{2}+\left(\frac{c}{1-c}\right)\frac{k+m}{2}\right]. \qquad [37]$$

Noting that

$$1-c = \left(\frac{1}{1+\frac{k}{m}f}\right)\left(\frac{1}{1+\frac{k}{m(1+\tau)}f}\right), \qquad [38]$$

further simplification leads to

$$m_1(f) \;=\; \left(\frac{k}{m}\right)^{k/2}\frac{1}{(\tau^2+1)^{(\frac{k}{2}+1)}}\left[\frac{1}{B\left(\frac{k}{2},\frac{m}{2}\right)}\frac{f^{k/2-1}}{\left[1+\frac{kf}{m(1+\tau^2)}\right]^{\frac{k+m}{2}}}\right. \qquad [39]$$

$$\left. +\; \frac{k\tau^2}{m(\tau^2+1)}\frac{1}{B\left(\frac{k}{2}+1,\frac{m}{2}\right)}\frac{f^{k/2}}{\left[1+\frac{kf}{m(1+\tau^2)}\right]^{\frac{k+m}{2}+1}}\right]. \qquad [40]$$

Substituting $\frac{k}{k+m}B(\frac{k}{2},\frac{m}{2})$ for $B(\frac{k}{2}+1,\frac{m}{2})$ and dividing $m_1(f)$ by $m_0(f)$ yields the Bayes factor specified in theorem.

■

As an aside, if

$$\frac{Y}{1+\tau^2} \sim F(k,m,0) \qquad \text{and} \qquad \frac{kZ}{(k+2)(1+\tau^2)} \sim F(k+2,m,0), \tag{41}$$

then

$$m_1(f) = \frac{1}{1+\tau^2}\, p_Y(f) + \frac{\tau^2}{1+\tau^2}\, p_Z(f). \tag{42}$$

Selection of τ^2

As stated in the main article, our criteria for selecting τ^2 in the specification of the prior distribution on the non-centrality parameters of the test statistics is to select τ^2 so that the prior modes on the non-centrality parameters correspond to a specified standardized effect size ω. For scalar variables, standardized effect sizes refer to the number of standard deviations the mean of a single observation falls from a specified null hypothesis, while for vector-valued parameters we refer to standardized effect size as the effect size standardized by the Cholesky decomposition of the (asymptotic) covariance matrix. Rationale for the choice of τ^2 for each of the tests listed in Table 1 follow.

One-sample z and t tests. Suppose x_i, $i = 1,\ldots,n$, represent independent and identically distributed (iid) $N(\mu,\sigma^2)$ random variables. Without loss of generality, the point null hypothesis may be specified as $H_0 : \mu = 0$ and either Theorem 1 (σ^2 known) or Theorem 2 (σ^2 unknown) may be applied, with test statistics $\sqrt{n}\bar{x}/\sigma$ or $\sqrt{n}\bar{x}/2$, respectively. In both cases, the non-centrality parameter is $\lambda = \sqrt{n}\mu/\sigma$. The modes of a $J(0,\tau^2)$ density are $\pm\sqrt{2}\tau$. Defining the standardized effect $\omega = \mu/\sigma$ and equating $\lambda^2 = 2\tau^2$ leads to $\tau^2 = n\omega^2/2$.

Two-sample z and t tests. Suppose x_{ij}, $i = 1,\ldots,n_j$, $j = 1,2$, represent iid $N(\mu_j,\sigma^2)$ random variables. The point null hypothesis is $H_0 : \mu_1 = \mu_2$ and either Theorem 1 (σ^2 known) or Theorem 2 (σ^2 unknown) may be applied, with test statistics

$$z = \frac{\bar{x_1} - \bar{x_2}}{\sigma\sqrt{1/n_1 + 1/n_2}} \qquad \text{or} \qquad t_\nu = \frac{\bar{x_1} - \bar{x_2}}{s\sqrt{1/n_1 + 1/n_2}}, \tag{43}$$

where $\nu = n_1 + n_2 - 2$.

The standardized effect size for both tests is $\omega = (\mu_1 - \mu_2)/(\sqrt{2}\sigma)$, and the non-centrality parameter is

$$\lambda = \frac{\mu_1 - \mu_2}{\sigma\sqrt{1/n_1 + 1/n_2}} = \frac{\sqrt{2n_1n_2}\omega}{\sqrt{n_1+n_2}}, \tag{44}$$

Equating $\lambda = \sqrt{2}\tau$ leads to

$$\tau^2 = \frac{n_1 n_2\, \omega^2}{n_1 + n_2}. \tag{45}$$

Multinomial and Poisson Tests. We assume that the asymptotic conditions described in, for example, (3) are satisfied for chi-squared test statistics of the form

$$h(\hat{\boldsymbol{\theta}}) = \sum_{k=1}^{K} \frac{[n_k - nf_k(\hat{\boldsymbol{\theta}})]^2}{nf_k(\hat{\boldsymbol{\theta}})}, \tag{46}$$

defined for counts n_k in K cells, an s dimensional parameter $\theta \in \Theta$ with $1 \le s < K$, and a $k \times 1$ vector-valued function $\mathbf{f}(\boldsymbol{\theta}) = (f_1(\boldsymbol{\theta}),\ldots,f_K(\boldsymbol{\theta}))'$ that satisfies $\sum_k f_k(\boldsymbol{\theta}) = 1$. Given $n = \sum_{k=1}^K n_k$, the counts $\{n_k\}$ are assumed to follow a multinomial distribution. In particular, this assumption is satisfied if the counts n_k arise as independent Poisson random variables. Under the null hypothesis, the cell probabilities satisfy $H_0 : \boldsymbol{\pi} = f_k(\boldsymbol{\theta})$ for some $\boldsymbol{\theta} \in \Theta$. Under the alternative hypothesis, the true cell probabilities $\mathbf{p}$ are assumed to satisfy $\mathbf{p} - \boldsymbol{\pi} = O(n^{-1/2})$. If $\hat{\boldsymbol{\theta}}$ is the maximum likelihood or minimum chi-squared estimate of $\boldsymbol{\theta}$, then under certain regularity conditions, (4) showed that $h(\hat{\boldsymbol{\theta}})$ converges to a non-central chi-squared distribution on $K - s - 1$ degrees of freedom and non-centrality parameter

$$n(\mathbf{p} - \boldsymbol{\pi})'\mathbf{D}_{\boldsymbol{\pi}}^{-1}(\mathbf{p} - \boldsymbol{\pi}), \quad \text{where} \quad \mathbf{D}_{\boldsymbol{\pi}} = \{\text{diag}(\boldsymbol{\pi})\}. \tag{47}$$

We define the standardized effect size vector as

$$\boldsymbol{\omega} = \left\{ \frac{p_k - \pi_k}{\sqrt{\pi_k}} \right\}. \tag{48}$$

The mode of the prior density on the non-centrality parameter in Theorem 3 occurs at $k\tau^2$, and the non-centrality parameter can be written as $n\boldsymbol{\omega}'\boldsymbol{\omega}$. Matching the mode of the prior density to the non-centrality parameter leads to

$$\tau^2 = \frac{n\boldsymbol{\omega}'\boldsymbol{\omega}}{k}. \tag{49}$$

In some applications, it is convenient to replace $\boldsymbol{\omega}'\boldsymbol{\omega}$ by $k\overline{\omega^2}$, where $\overline{\omega^2} = \frac{1}{k}\sum_{i=1}^k \omega_i^2$, the average squared standardized effect.

Linear Models. We assume that the $n \times 1$ data vector $\mathbf{y}$ satisfies

$$\mathbf{y} \sim N(\mathbf{X}\boldsymbol{\beta}, \sigma^2 \mathbf{I}_n) \qquad [50]$$

for a $p \times 1$ vector $\boldsymbol{\beta}$ and $n \times p$ matrix $\mathbf{X}$ of rank $p < n$. The null hypothesis can be expressed as $H_0 : \mathbf{A}\boldsymbol{\beta} = \mathbf{a}$ where the rank of $\mathbf{A}$ is $k \leq p$. The F statistic against the alternative hypothesis $H_1 : \mathbf{A}\boldsymbol{\beta} \neq \mathbf{a}$ can be expressed as

$$F_{k,n-p} = \frac{(RSS_0 - RSS_1)/k}{RSS_1/(n-p)}, \qquad [51]$$

where RSS_0 is the constrained residual sum-of-squares under the null model and RSS_1 is the residual sum-of-squares under the unconstrained model.

As demonstrated in, for example (5), the non-centrality parameter for the F statistic can be expressed as

$$\lambda = \frac{n(\mathbf{A}\boldsymbol{\beta} - \mathbf{a})'\mathbf{V}^{-1}(\mathbf{A}\boldsymbol{\beta} - \mathbf{a})}{2\sigma^2}, \qquad \text{where} \quad \mathbf{V} = \left[\mathbf{A} \left(\frac{\mathbf{X}'\mathbf{X}}{n} \right)^{-1} \mathbf{A}' \right]. \qquad [52]$$

Letting $\mathbf{L}$ denote the Cholesky decomposition of $\mathbf{V}$, i.e., $\mathbf{L}\mathbf{L}' = \mathbf{V}$, we define the standardized effect size vector $\boldsymbol{\omega}$ as

$$\boldsymbol{\omega} = \frac{\mathbf{L}^{-1}(\mathbf{A}\boldsymbol{\beta} - \mathbf{a})}{\sigma}. \qquad [53]$$

It follows that the non-centrality parameter can be written

$$\lambda = \frac{n\boldsymbol{\omega}'\boldsymbol{\omega}}{2}. \qquad [54]$$

Equating $\lambda = k\tau^2$ leads to

$$\tau^2 = \frac{n\boldsymbol{\omega}'\boldsymbol{\omega}}{2k}. \qquad [55]$$

As for count models, it is sometimes convenient to replace $\boldsymbol{\omega}'\boldsymbol{\omega}$ by $k\overline{\omega^2}$, where $\overline{\omega^2} = \frac{1}{k}\sum_{i=1}^{k} \omega_i^2$, the average squared standardized effect.

Likelihood Ratio Statistic. We assume that the $n \times 1$ data vector $\mathbf{y}$ is generated from a parametric family of densities indexed by $\boldsymbol{\theta} = (\boldsymbol{\theta}_r, \boldsymbol{\theta}_s)$, say $f(\mathbf{y}, \boldsymbol{\theta})$. The likelihood function is denoted by $L(\boldsymbol{\theta} \,|\, \mathbf{y})$. The null hypothesis is $H_0 : \boldsymbol{\theta}_r = \boldsymbol{\theta}_{r0}$, where $\dim(\boldsymbol{\theta}_r) = k$. The likelihood ratio statistic is

$$h = -2 \log \left[\frac{L(\boldsymbol{\theta}_{r0}, \hat{\boldsymbol{\theta}}_s)}{L(\hat{\boldsymbol{\theta}})} \right], \qquad [56]$$

where $\hat{\boldsymbol{\theta}}$ is the unconstrained maximum likelihood estimate (MLE) and $\hat{\boldsymbol{\theta}}_s$ is the constrained MLE under H_0.

Assuming regularity conditions in (6), h converges to a χ^2 distribution on k degrees of freedom and non-centrality parameter

$$\lambda = n(\boldsymbol{\theta}_r - \boldsymbol{\theta}_{r0})'\mathbf{V}_r^{-1}(\boldsymbol{\theta}_r - \boldsymbol{\theta}_{r0}) \qquad \text{where} \qquad V_{r_i,r_j}^{-1} = -\frac{1}{n} E\left[\frac{\partial^2 \log L}{\partial\theta_{r_i}\partial\theta_{r_j}} \right]. \qquad [57]$$

Let $\mathbf{L}$ denote the Cholesky decomposition of $\mathbf{V}_r$ and define

$$\boldsymbol{\omega} = \mathbf{L}^{-1}(\boldsymbol{\theta}_r - \boldsymbol{\theta}_{r0}). \qquad [58]$$

Then $\lambda = n\boldsymbol{\omega}'\boldsymbol{\omega}$, and setting $\lambda = k\tau^2$ implies

$$\tau^2 = \frac{n\boldsymbol{\omega}'\boldsymbol{\omega}}{k}. \qquad [59]$$

For independent and identically distributed observations, $\mathbf{V}_r^{-1}$ has elements

$$V_{r_i,r_j}^{-1} = -E\left[\frac{\partial^2 \log f}{\partial\theta_{r_i}\partial\theta_{r_j}} \right]. \qquad [60]$$

If $\mathbf{L}$ is instead defined from the information matrix $\mathbf{V}_r$ for a single observation, then the standardized effect size can be redefined as

$$\boldsymbol{\omega} = \mathbf{L}^{-1}(\boldsymbol{\theta}_r - \boldsymbol{\theta}_{r0}). \qquad [61]$$

Further examples of z tests

The examples in the main article depicted BFFs in which there was positive support for alternative hypotheses centered on some subset of standardized effect sizes. In this section, we briefly examine the shape of BFF curves when data provide no or little support for any alternative hypotheses. For simplicity, we restrict attention to z tests of a normal mean with a sample size $n = 10,000$. The figure below depicts BFFs for z statistics ranging from 0 to 2.5.

Figure S1 shows that for large values of n (e.g., $n = 10,000$), BFFs decrease from 1 as the standardized effect increases for $z \leq 1$, but still exhibit maximum values greater than 1.0 for $z > 1$. The BFF never exceeds 1:1 for any effect size when $z \leq 1$.

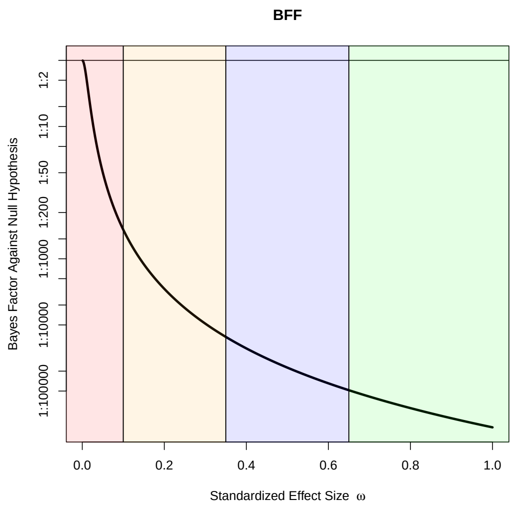

(a) $z = 0.0$

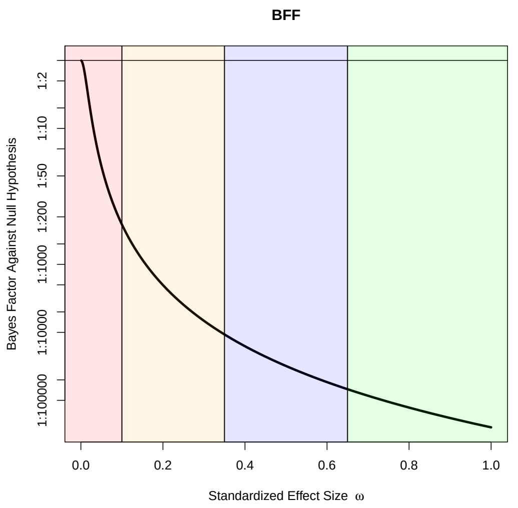

(b) $z = 0.5$

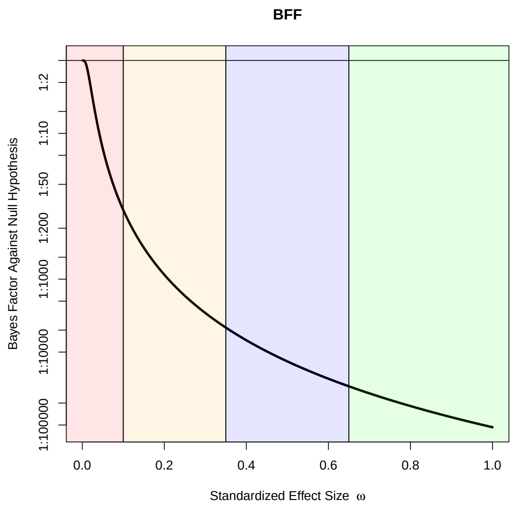

(c) $z = 1.0$

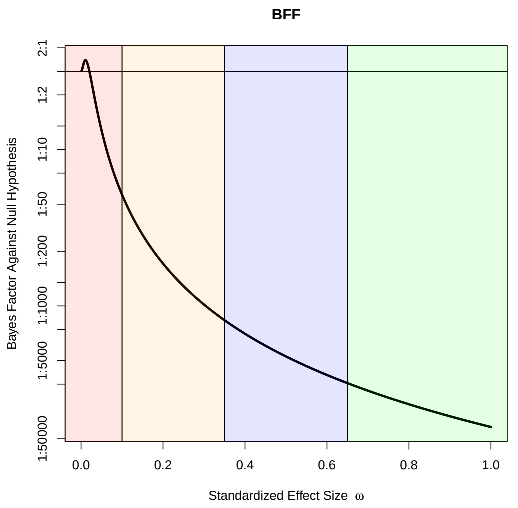

(d) $z = 1.5$

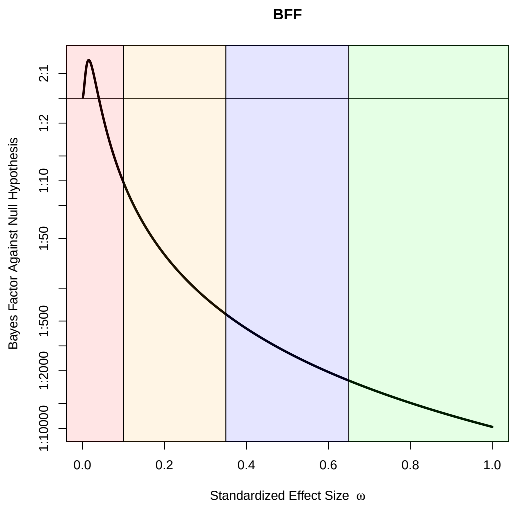

(e) $z = 2.0$

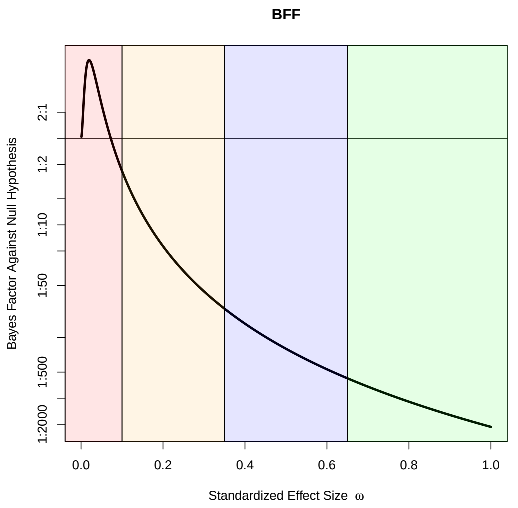

(f) $z = 2.5$

Fig. S1. BFF curves for z statistics obtained for a sample size of $n = 10,000$.

References

1. S Pramanik, VE Johnson, Efficient alternatives for Bayesian hypothesis tests in psychology. *Psychol. Methods* (2023).
2. M Abramowitz, IA Stegun, *Handbook of Mathematical Functions with Formulas, Graphs and Mathematical Tables.* (Dover, New York), Ninth edition, (1970).
3. YM Bishop, SE Feinberg, PW Holland, *Discrete Multivariate Analysis: Theory and Practice.* (MIT Press, Cambridge), (1975).
4. S Mitra, On the limiting power function of the frequency chi-square test. *Annals Math. Stat.* **29**, 1221–1233 (1958).
5. R Hocking, *The Analysis of Linear Models.* (Brooks/Cole, Monterey, CA), (1985).
6. A Stuart, JK Ord, *Kendall's Advance Theory of Statistics.* (Oxford University Press, New York) Vol. 2, (1991).
